# Turnover rate of tear-film lipid layer determined by fluorophotometry

**DOI:** 10.1136/bjo.2008.156828

**Published:** 2009-08-18

**Authors:** H Mochizuki, M Yamada, S Hatou, K Tsubota

**Affiliations:** 1 Division for Vision Research, National Institute of Sensory Organs, National Tokyo Medical Center, Tokyo, Japan; 2 Department of Ophthalmology, Keio University School of Medicine, Tokyo, Japan

## Abstract

**Aim:**

This study was performed to independently assess the turnover rates of aqueous and lipid layers of the tear film.

**Methods:**

Two fluorescent dyes, fluorescein sodium and 5-dodecanoylaminofluorescein (DAF), which is a free-fatty-acid conjugate of fluorescein, were applied to the right eye of 12 healthy volunteers. Fluorescent intensity of the precorneal tear film was measured at the central cornea every minute for 10 min for fluorescein sodium, and every 5 min for 50 min for DAF. The turnover rate was calculated by plotting fluorescent intensity against time in a semilog plot and expressed as %/min.

**Results:**

Turnover rates of fluorescein sodium and DAF were 10.3 (SD 3.7)%/min and 0.93 (0.36)%/min, respectively. The turnover rate of DAF was significantly lower than that of fluorescein sodium (p<0.05, Mann–Whitney test). The turnover rate of DAF positively correlated with that of fluorescein sodium (r = 0.93, p<0.05).

**Conclusion:**

Our results indicate that the turnover of lipids in tears is much slower than the aqueous flow of tears, and that this lipid turnover is associated with the aqueous flow of tears in healthy adults.

The precorneal tear film has traditionally been described as consisting of an outer lipid layer, a middle aqueous layer and an inner mucus layer. Although this remains valid, some modifications have been proposed.^1^ ^2^ ^3^ In the current model of the tear film, the aqueous-mucin layer is covered by two thin layers of lipids. Polar lipids such as phospholipids lie adjacent to the aqueous-mucin layer, and non-polar lipids such as cholesterol and wax ester are present at the tear–air interface. In addition, tears contain proteins that possess lipid-binding properties, such as tear lipocalin.^4^ ^5^ ^6^ Although lipids in tears are primarily located in the tear-film lipid layer, some lipids are presumably bound by lipocalin in the aqueous layer. Tear lipocalin is thought to have an important role in stabilising the tear-film lipid layer by transferring lipids to it from the aqueous layer.^4^ ^5^ ^6^

Despite comprising a very small proportion of the overall tear-film thickness, the lipid layer is important for retarding evaporation and maintaining tear-film stability.^2^ ^3^ Where the lipid layer is absent or where the integrity of the lipid layer is compromised, the evaporation rate of tears increases, accompanied by tear-film instability.^7^ ^8^ To assess the lipid layer of tears, several techniques have been developed, including observation of lipid layer characteristics by interferometric methods,^9^ ^10^ ^11^ quantitative measurement of meibomian lipid on the lid margin by meibometry^12^ ^13^ and measurement of evaporation from the ocular surface.^14^ ^15^ ^16^ Of these, observation of lipid layer characteristics by interferometric methods has been well established.^9^ ^10^ ^11^ ^17^ In various pathological conditions, such as meibomian gland dysfunction, the appearance of the lipid layer can change. Lipid layer thickness, measured by interferometry, has been reported to correlate with tear-film evaporation, tear-film breakup time, and clinical symptoms.^8^ ^18^ We have previously reported that the concentration of lipocalin in tears from patients with meibomian gland dysfunction was significantly lower than in normal controls.^19^ Thus, lipids in tears, both in the lipid layer and in the aqueous layer held by lipocalin, are important when considering the pathophysiology of evaporative dry eye, such as meibomian gland dysfunction. Until now, however, there has been no information about the flow rate of tear-film lipid layer.

Aqueous tear flow is determined by several aspects of tear dynamics including tear production, tear volume, tear evaporation and tear outflow.^20^ Tear flow can be assessed by introducing a dye or radioactive substance into the conjunctival sac and measuring the decay in concentration over a certain period. Since the report of Mishima *et al*,^21^ fluorophotometric measurement using fluorescein sodium as a tracer has been the gold standard to quantify tear flow.^16^ ^20^ The elimination rate of fluorescein sodium essentially represents the bulk aqueous flow because the dye is hydrophilic; however, the turnover of a certain tear component may not parallel the bulk aqueous flow. For example, we recently reported differences between the bulk aqueous flow of hyaluronic acid and the turnover of hyaluronic acid, suggesting that hyaluronic acid remains on the ocular surface independent of the bulk aqueous flow.^22^ Accordingly, we hypothesised that the flow rate of the tear lipid layer might be different from that of aqueous tear layer.

In this study, we tested this hypothesis using fluorescein sodium and a free-fatty-acid conjugate of fluorescein. Fluorescein was used to assess the aqueous flow, and the conjugated dye was used as a tracer to determine the flow rate of the tear lipid layer.

## Methods

### Fluorescent dye and fluorophotometer

5-Dodecanoylaminofluorescein (DAF; Molecular Probes, Eugene, Oregon) is a lipophilic and water-insoluble free-fatty-acid conjugate of fluorescein. This dye has the longest-wavelength absorption maximum at 495 nm, and an emission spectrum that peaks at 518 nm. A DAF emulsion (50 mg/ml) was prepared in sterile 0.067 M phosphate-buffered saline (PBS), pH 7.4, with 1% Tween 80 (Sigma-Aldrich, St Louis, Missouri). Fluorescein sodium (Sigma-Aldrich) was dissolved in sterile 0.067 M PBS and used as a tracer of the tear aqueous layer. No signs of inflammation or damage were detected either immediately or after 24 h by instilling five drops of 5% DAF emulsion at 10 min intervals into four rabbits’ eyes. Instillation of one drop of 5% DAF emulsion into the eyes of four subjects caused no discomfort, and no staining or adverse effects were detected by a slit-lamp examination. To test the effect of DAF emulsion on tear-film stability, we measured tear-film break-up time (BUT) after instilling a 1 μl of DAF emulsion (50 mg/ml). The DAF-BUT was 21.6 (10.4) s (n = 20), which was longer than fluorescein BUT (13.1 (3.6) s) measured by instilling 1 μl of fluorescein sodium solution (50 μg/ml) on a different day. From these preliminary experiments, a DAF emulsion was considered to have no apparent adverse effects on the ocular surface or the tear film.

**Figure 1:**
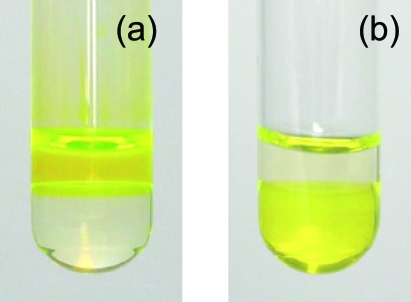
Fluorescein sodium (A) and 5-dodecanoylaminofluorescein (DAF) (B) after a modified Bligh and Dyer procedure. The fluorescent yellowish colour of fluorescein sodium was seen in the upper aqueous layer, whereas the dye colour of DAF was seen in the lower lipid layer.

A modified Bligh and Dyer procedure was performed to test the nature of these dyes. Ten microlitres of 5% DAF emulsion and 0.5% fluorescein sodium solution were placed in a test tube with 1 ml of a 2:1 chloroform:methanol solvent (Wako, Osaka, Japan). After adding 0.4 ml of water, the tubes were vortexed for 30 s. The solvent formed two layers—an upper aqueous layer and a lower lipid layer. As expected, the fluorescent yellowish colour of DAF was seen in the lower lipid layer, whereas the dye colour of fluorescein sodium was seen in the upper aqueous layer (fig 1). Spectrophotometric measurements (495 nm) revealed that 99% of DAF was located in the lower lipid layer, and 97% of fluorescein sodium was in the upper aqueous layer.

A commercial slit-lamp fluorophotometer (Anterior Fluorometer FL-500, Kowa, Tokyo) was used to quantify fluorescence intensity. The illuminating light was focused as a 2 mm diameter circle on the surface of the cornea. The emitted light passed through a band interference filter centred on 565 nm (half bandwidth 25 nm) and was directed to a photomultiplier tube with the band interference filter centred on a wavelength of 490 nm (half bandwidth 30 nm).

DAF emulsion (50 mg/ml) was diluted in PBS to produce standards ranging from 0.01 to 50 mg/ml for calibration. Fluorescein sodium solution (5 mg/ml) was diluted to produce standards ranging from 0.1 to 50 μg/ml in the same fashion. A cuvette was constructed by gluing together two microscopic slides and two cover glasses. The cover glasses were sandwiched by the two slides to provide a 12–15 μm thick space for the fluid layer. A fresh cuvette was used for each solution. The standards (10 μl) were added to the cuvettes, and fluorescence intensity was measured by the slit-lamp fluorophotometer. The interaction of DAF or fluorescein sodium with proteins was also tested by diluting the standards in PBS containing 10% fetal bovine serum (FBS).

### Measurement of turnover rate

Twelve healthy volunteers (six males and six females) aged 21 to 47 years (mean 32.6 (SD 8.2) years), who had no history of eye disease except for refractive errors, were enrolled in the study. One of the authors (SH) performed a routine ocular examination on all subjects, followed by an examination of the ocular surface, including Schirmer testing and measurement of tear-film break-up time (BUT). For vital staining, 2 μl of a saline solution containing 1% fluorescein was used. All subjects had more than 5 mm of Schirmer strip wetting, more than 5 s in tear-film BUT, and no apparent fluorescein staining of cornea and conjunctiva. The Marx lines, which run along the eyelid margin determined by fluorescein staining, were normal in all subjects.^23^ The guidelines of the World Medical Association Declaration of Helsinki were followed. The subjects received a full explanation of the procedures and provided their informed consent for participation prior to the experiment. The protocol was approved by the institutional review board of National Tokyo Medical Center (R-07-011: Assessment of layer-by-layer tear dynamics by fluorophotometry), and all subjects provided written informed consent.

The subjects were seated in front of the fluorophotometer. The instrument was focused on the central cornea, and background fluorescence intensity was measured. A volume of 1 μl of DAF emulsion (50 mg/ml) or fluorescein sodium solution (50 μg/ml) was applied to the right eye using an Eppendorf micropipette without making contact with the ocular surface. The subjects were instructed to blink several times to ensure mixing of the dye. Fluorescence intensity of the precorneal tear film was measured at the central cornea. When fluorescein sodium solution was instilled into the eye, fluorescence intensity was measured every minute for 10 min. When DAF emulsion was instilled, measurements were repeated every 5 min for 50 min because of a slower decay of intensity. Measurements of fluorescein sodium and DAF were done on different days. Measurements of DAF were repeated on three different days in three subjects to evaluate the repeatability of the test.

The turnover rate was determined by plotting fluorescence intensity against time in a semilog plot, F = F_0_exp(−kt), where F = fluorescence intensity at time (t), F_0 = _fluorescence intensity at time zero, k = turnover rate and t = time (min). The turnover rate was calculated for all tests and expressed as %/min. The regression fit of the log of fluorescence intensity was recorded as the regression coefficient.

When DAF values were plotted, the regression line was straight. On the other hand, when fluorescein sodium values were plotted, some regression lines were biphasic, representing an initial faster turnover rate and a subsequent slower turnover rate. In cases in which the turnover rate of fluorescein sodium became biphasic, the subsequent slower turnover rate was used as the flow rate.

All results are presented as the mean (SD). Statistical significance was calculated by comparing results using the Mann–Whitney test. A p value of <0.05 was considered statistically significant.

## Results

### Calibration of DAF and fluorescein sodium

**Figure 2:**
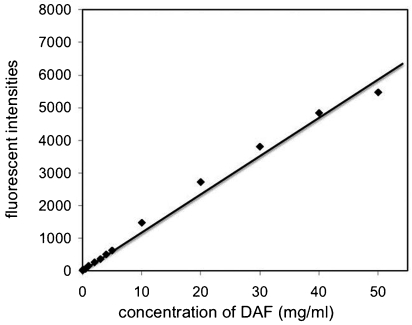
Plot of fluorescence intensity and 5-dodecanoylaminofluorescein (DAF) concentration. The relationship between fluorescence intensity and DAF concentration was linear (r^2^ = 0.991).

The relationship between fluorescence intensity and DAF concentration was linear (fig 2, r^2^ = 0.991). The data generated by this method were consistent and reproducible. Fluorescence intensity was unaffected by the presence of 10% FBS (data not shown). Similar results were obtained when the fluorescein sodium standards were tested (data not shown).

### Turnover rate

**Figure 3:**
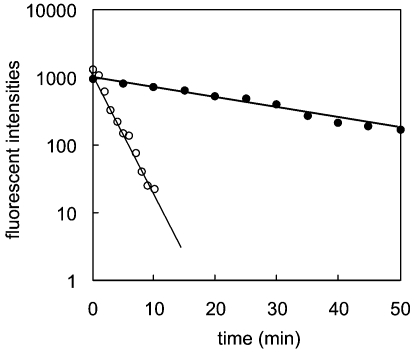
Representative result of turnover rate obtained from one subject. Fluorescence intensity of 5-dodecanoylaminofluorescein (black circle) decayed with time at a flow rate of 1.14%/min, which was lower than that of fluorescein sodium (14.5%/min; white circle).

A representative result of turnover rate obtained from one subject is shown in fig 3. Fluorescence intensity of fluorescein sodium decayed with time at a flow rate of 14.5%/min. In the presented case, the fluorescence decay rate of DAF was much lower (1.14%/min) that that of fluorescein sodium. To test the reproducibility of the DAF method, the measurement was repeated three times in three eyes of three subjects. The coefficient of variance of the measurements was lower than 0.1 in all cases (mean = 0.07, n = 3), which ensured the reproducibility of the measurement.

The turnover rates of DAF and fluorescein sodium were 0.93 (0.36)%/min and 10.3 (3.7)%/min, respectively (table 1). The turnover rate of DAF was significantly lower than that of fluorescein sodium (p<0.05).

**Table 1:** Turnover rates of a topically applied (1 μl) 50 mg/ml 5-dodecanoylaminofluorescein (DAF) emulsion or 50 μg/ml fluorescein sodium solution observed in 12 subjects

Subject no	Turnover rate (%/min)	
	DAF	Fluorescein sodium
1	0.51	5.2
2	0.46	4.4
3	0.54	7.1
4	0.61	7.2
5	0.84	9.7
6	0.93	10.0
7	0.84	10.2
8	1.12	11.5
9	1.60	14.7
10	1.31	12.9
11	1.24	15.8
12	1.14	14.5
Mean (SD)	0.93 (0.36)	10.3 (3.7)

The turnover rate of DAF was significantly lower than that of fluorescein sodium (p<0.05).

**Figure 4:**
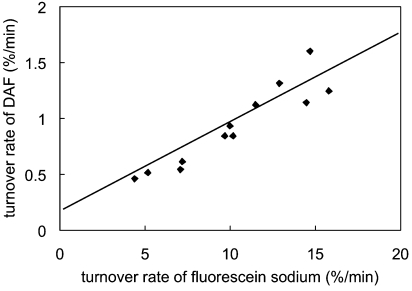
Relationship between the turnover rates of 5-dodecanoylaminofluorescein (DAF) and fluorescein sodium. The DAF turnover rate positively correlated with that of fluorescein sodium (r^2^ = 0.87, p<0.05).

The turnover rate of DAF positively correlated with that of fluorescein sodium (fig 4; r^2^ = 0.87, p<0.05).

## Discussion

We used fluorescein sodium and DAF to assess independently the turnover rates of aqueous and lipid layers of the tear film. To the best of our knowledge, this is the first study to address the turnover rate of the tear-film lipid layer.

There are potential limitations when interpreting the results of the present study, of which one is that DAF is a free-fatty-acid conjugate of fluorescein. Tear lipids are known to consist of various classes of lipids, such as wax esters, cholesterol, cholesterol esters, phospholipids, glycolipids and free fatty acids.^2^ ^3^ ^24^ Because different classes of lipids have different biophysical properties, including molecular weight, hydrophobicity, viscosity and binding capacity to tear lipocalin,^24^ ^25^ the turnover of all lipids in tears may not be the same as that of DAF. Another limitation is the usage of a 5% DAF emulsion containing Tween 80. Although the addition of a surfactant was essential to make the emulsion of DAF, we considered it undesirable because it might disrupt the tear-film stability. We minimised the amount of Tween 80 in the emulsion, and the applied volume of DAF emulsion into the eye, to avoid the effect of a surfactant as much as possible.

The most interesting finding was that the turnover rate of DAF (0.9 (0.4)%/min) was approximately 9% that of fluorescein sodium (10.3 (3.8)%/min), indicating that the turnover of lipids in tears is much slower than the aqueous flow of tears. The discrepancy between the results of the dyes may be explained by the small bulk of the tear-film lipid layer compared with that of the marginal reservoirs.^2^ Using meibometry, Chew *et al*^12^ ^13^ estimated that approximately 300 μg of lipid is present in the marginal reservoir and calculated that the preocular tear film contains approximately 9 μg of lipids. It has been estimated that the volume of the preocular tear film is 1–1.5 μl and that the total volume of tear fluid is 7–10 μl.^20^ Our results confirm that the lipid layer, despite comprising a very small proportion of the overall tear film, is a distinct component of the tear film from others.

Another important finding of our study is that the turnover rate of DAF correlates well with that of fluorescein sodium. This result indicates that subjects with a high turnover rate of aqueous tears tend to have a high turnover rate of lipids in tears. Until now, the mechanism for excretion of tear lipids has not been fully understood. Bron *et al*^2^ stated that excretion most likely occurs by bulk flow over the lid margin and onto the neighbouring lid skin and lashes. They also indicated that lipids may be excreted by diffusion from the tear-film lipid layer into the aqueous phase of the tear film. In the latter, the biochemical interaction between proteins and lipids may have a role in transferring and scavenging lipids in tears.^2^ ^4^ ^5^ ^6^ Our results suggest that lipid turnover in tears is, at least partially, associated with the aqueous flow of tears. The association between them may be due to the facilitated excretion onto the lid skin, because a higher aqueous flow of tears is sometimes associated with a larger tear volume.^16^ Alternatively, the association may reflect the turnover of a lipid fraction bound by lipocalin in the aqueous layer.^4^ ^5^ ^6^ Our current methodology, however, is not able to distinguish two routes of lipid excretion from tears.

The data presented in the current study were obtained from healthy adults. Lipid turnover in tears may be different in older subjects or in those with meibomian gland dysfunction. The anterior displacements of muco-cutaneous junction are associated with ageing and the presence of meibomian gland dysfunction.^23^ In this situation, some meibomian gland orifices are open posterior to the muco-cutaneous junction. Therefore, the dynamics of tear lipids excretions in these cases may differ from normal subjects. Further investigations have been planned to clarify these issues.
